# Follistatin-Like 1: A Potential Mediator of Inflammation in Obesity

**DOI:** 10.1155/2013/752519

**Published:** 2013-11-21

**Authors:** Nengguang Fan, Haiyan Sun, Yufan Wang, Yifei Wang, Lijuan Zhang, Zhenhua Xia, Liang Peng, Yanqiang Hou, Weiqin Shen, Rui Liu, Jiajing Yin, Yongde Peng

**Affiliations:** ^1^Department of Endocrinology, Shanghai First People's Hospital, Shanghai Jiao Tong University, 100 Haining Road, Shanghai 200080, China; ^2^Department of Endocrinology, Shanghai Songjiang Center Hospital, 746 Zhongshan Zhong Road, Shanghai 201600, China; ^3^Department of Laboratory Medicine, Shanghai Songjiang Center Hospital, 746 Zhongshan Zhong Road, Shanghai 201600, China; ^4^Department of Medical Examination, Shanghai Songjiang Center Hospital, 746 Zhongshan Zhong Road, Shanghai 201600, China

## Abstract

Obesity is associated with a state of chronic low-grade inflammation, which contributes to insulin resistance and type 2 diabetes. However, the molecular mechanisms that link obesity to inflammation are not fully understood. Follistatin-like 1 (FSTL1) is a novel proinflammatory cytokine that is expressed in adipose tissue and secreted by preadipocytes/adipocytes. We aimed to test whether FSTL1 could have a role in obesity-induced inflammation and insulin resistance. It was found that FSTL1 expression was markedly decreased during differentiation of 3T3-L1 preadipocytes but reinduced by TNF-**α**. Furthermore, a significant increase in FSTL1 levels was observed in adipose tissue of obese ob/ob mice, as well as in serum of overweight/obese subjects. Mechanistic studies revealed that FSTL1 induced inflammatory responses in both 3T3-L1 adipocytes and RAW264.7 macrophages. The expression of proinflammatory mediators including IL-6, TNF-**α**, and MCP-1 was upregulated by recombinant FSTL1 in a dose-dependent manner, paralleled with activation of the IKK**β**-NF**κ**B and JNK signaling pathways in the two cell lines. Moreover, FSTL1 impaired insulin signaling in 3T3-L1 adipocytes, as revealed by attenuated phosphorylation of both Akt and IRS-1 in response to insulin stimulation. Together, our results suggest that FSTL1 is a potential mediator of inflammation and insulin resistance in obesity.

## 1. Introduction

Obesity has reached epidemic proportions worldwide and affects both adults and children [1]. Accumulating evidence indicates that obesity is associated with chronic low-grade inflammation, characterized by increased proinflammatory cytokine production and accumulation of macrophages in adipose tissue [2–4]. Initially viewed as a major site for energy storage, recently it has become evident that adipose tissue is also an important endocrine and immune organ, which secretes a variety of bioactive molecules termed as adipokines [5]. During the development of obesity, the secretory function of adipose tissue changes dramatically. Whereas the secretion of proinflammatory cytokines such as TNF-*α* and IL-6 is increased, the production of anti-inflammatory adipokines/cytokines is reduced [6, 7]. The imbalance between pro- and anti-inflammatory mediators contributes to the chronic inflammation in obesity, which in turn leads to insulin resistance and other obesity-associated diseases [6, 7]. To date, many proinflammatory cytokines, in addition to TNF-*α* and IL-6, have been linked to obesity-induced inflammation and insulin resistance [6–8].

Follistatin-like 1 (FSTL1) is a secreted extracellular glycoprotein, which has recently been identified as a novel proinflammatory cytokine [9]. It was originally cloned from an osteoblast cell line as a TGF-*β*-inducible gene [10]. Based on sequence homology, FSTL1 belongs to the BM/SPARC/osteonectin family that contains both extracellular calcium-binding and follistatin-like domains [11]. However, the calcium-binding domains of FSTL1 are nonfunctional [11], suggesting that FSTL1 may have distinct functional features. With a wide expression pattern (e.g., lung, heart, brain, and urinary tract), FSTL1 has shown diverse and cell type-specific functions, including the regulation of cell proliferation, apoptosis, differentiation, and migration [12–14]. Consequently, FSTL1 is involved in multiple biological processes, such as angiogenesis, tumorigenesis, and embryonic development [13–16].

Recently, the emerging role of FSTL1 in inflammation has become evident. FSTL1 is overexpressed in synovial tissue of rheumatoid arthritis [17], and its serum levels are significantly elevated in patients with systemic inflammatory diseases, such as rheumatoid arthritis, ulcerative colitis, and systemic lupus erythematosus [18]. Animal experiments further demonstrated a causative role of FSTL1 in inflammation [9, 17]. Adenovirus-mediated administration of FSTL1 to mice exacerbated collagen-induced arthritis [9], while its neutralization with specific antibody was ameliorative [17]. Notably, FSTL1 can induce inflammation in liver [9], where it is not normally expressed [19]. In vitro, FSTL1 triggers inflammatory responses in both immune and nonimmune cells. Transfection of Fstl1 into macrophages and fibroblasts induced the secretion of TNF-*α*, IL-1*β*, and IL-6 [9]. In addition, recombinant FSTL1 increased IFN-*γ* production by T cells [17]. Conversely, targeted inhibition of FSTL1 in ST2 stromal cells ablated the secretion of IL-6 and MCP-1 induced by TNF-*α* plus IL-17 [20]. The proinflammatory actions of FSTL1 may be through activation of toll-like receptor 4 (TLR4) signaling [21]. Together, FSTL1 appears to be an important endogenous mediator of inflammation and is involved in many chronic inflammatory diseases. However, the role of FSTL1 in obesity-associated inflammation has not previously been studied.

Indeed, FSTL1 is expressed in adipose tissue of mice, mainly by the stromal vascular factions [19]. It is also synthesized and secreted by 3T3-L1 preadipocytes, and its level declines during in vitro adipogenesis [19]. In addition, as an inflammatory cytokine [17], FSTL1 seems to be closely associated with inflammatory states of adipocytes. Treatment of 3T3-L1 adipocytes with TNF-*α* induced the expression of FSTL1 [19]. Moreover, coculture of adipocytes with macrophages also upregulated FSTL1 expression in adipocytes [22]. However, the pathophysiological significance of FSTL1 expression in adipocytes in response to inflammatory stimulation remains unknown.

In light of the inflammatory induction of FSTL1 in adipocytes and its proinflammatory actions, we hypothesized that FSTL1 may be implicated in adipose tissue inflammation and insulin resistance in obesity. To address this hypothesis, we assessed FSTL1 expression levels in adipose tissue of obese mice, as well as in serum of overweight/obese subjects. In addition, we examined the proinflammatory effects of recombinant FSTL1 on both adipocytes and macrophages. Finally, the impact of FSTL1 on insulin signaling in adipocytes was determined.

## 2. Materials and Methods

### 2.1. Chemicals and Reagents

Dulbecco's modified Eagle's medium (DMEM) was purchased from Hyclone (Beijing, China). Fetal bovine serum (FBS) was from Gibco (Carlsbad, CA). Isobutylmethylxanthine, dexamethasone, insulin, and rosiglitazone were from Sigma (St. Louis, MO). Recombinant mouse FSTL1 was obtained from R&D Systems (Minneapolis, MN) and the endotoxin level was below 1.0 EU per 1 *μ*g of the protein by the LAL method. Human TNF-*α* was from Peprotech (Rocky Hill, NJ). Specific antibodies against p65, phospho-p65 (Ser536), phospho-IKK*β* (Ser181), JNK, phospho-JNK (Thr183/Tyr185), Akt, phospho-Akt (Ser473), insulin receptor substrate 1 (IRS-1), and GAPDH were purchased from Cell Signaling Technology (Beverly, MA). Antibodies against FSTL1 and phospho-IRS-1 (Tyr612) were from Abcam (Cambridge, MA). Horseradish-peroxidase- (HRP-) conjugated antibodies against rabbit or goat IgG were from Jackson Laboratories (West Grove, PA).

### 2.2. Subjects

A total of 144 subjects were consecutively recruited from individuals who visited the Medical Examination Center of Shanghai First People's Hospital for routine health checkups. Based on body mass index (BMI), subjects were divided into two groups: normal weight subjects (NW; BMI < 25 kg/m^2^, *n* = 93) and overweight/obese subjects (OW/OB; BMI ≥ 25 kg/m^2^, *n* = 51). Those with diabetes, acute or chronic infectious disease, autoimmune diseases, heart failure, or hepatic or renal disease were excluded. The study was approved by the Institutional Review Board of Shanghai First People's Hospital affiliated to Shanghai Jiao Tong University School of Medicine and performed in accordance with the principle of the Helsinki Declaration II. Written informed consent was obtained from all subjects.

### 2.3. Anthropometric and Biochemical Measurements

All subjects were assessed after overnight fasting. Body weight, height, systolic pressure (SBP), and diastolic pressure (DBP) were measured by an experienced physician. BMI was calculated as weight in kilograms divided by height in meters squared. Fasting blood glucose (FBG), triglycerides (TG), total cholesterol (TC), low-density lipoprotein cholesterol (LDL-C), and high-density lipoprotein cholesterol (HDL-C) were measured using an autoanalyser (Beckman). Serum FSTL1 was determined with a commercially available enzyme-linked immunosorbent assay (ELISA) kit (DuoSet, R&D Systems, Minneapolis, MN). The linear range of the assay was 0.325–10 ng/mL.

### 2.4. Animals

Male C57BL/6J leptin-deficient (ob/ob) mice and lean littermates (6 weeks of age) were purchased from the Model Animal Research Center of Nanjing University (Nanjing, China). Mice were housed in a pathogen-free barrier facility with a 12 h light/12 h dark cycle and given free access to standard chow diet and water. At 16 weeks of age, mice were sacrificed under sodium pentobarbital anesthesia. Subcutaneous and epididymal fat pads were snap-frozen in liquid nitrogen immediately after resection and stored at −80°C until use. All procedures conducted were approved by the Committee on the Ethics of Animal Experiments of Shanghai Jiao Tong University.

### 2.5. Cell Culture and Treatment

3T3-L1 preadipocytes and RAW264.7 macrophages were obtained from American Type Culture Collection (Rockville, MD) and maintained in DMEM supplemented with 10% FBS, 100 U/mL penicillin, and 100 *μ*g/mL streptomycin in a 5% CO_2_ humidified atmosphere at 37°C. Differentiation of 3T3-L1 preadipocytes was performed as described previously [23]. Briefly, 2 days after confluence (defined as D0), cells were exposed to differentiation medium containing 0.5 mM isobutylmethylxanthine, 1 *μ*M dexamethasone, 1.67 *μ*M insulin (MDI), and 10% FBS. After 48 h of incubation (D2), the medium was replaced with DMEM containing 10% FBS and 1.67 *μ*M insulin. On D4, the cells were switched to DMEM containing 10% FBS and refed every other day for the following 4–6 days until more than 90% of cells demonstrated adipocyte phenotype. 

Before FSTL1 treatment, fully differentiated 3T3-L1 adipocytes or RAW264.7 macrophages were serum starved in DMEM containing 0.25% FBS for 16 h and then stimulated with vehicle control or recombinant mouse FSTL1 for indicated time periods. For insulin signaling study, differentiated 3T3-L1 adipocytes were incubated with recombinant FSTL1 for 24 h and then stimulated with insulin (100 nM) for 10 min. Phosphorylation of Akt and IRS-1 were determined by Western blot analysis.

### 2.6. RNA Preparation and Quantitative Real-Time PCR Analysis

Total RNA was extracted from adipose tissue or cells with TRIzol Reagent (Invitrogen, Carlsbad, CA) according to the manufacturer's instructions. Subsequently, 1 *μ*g of total RNA was reverse-transcribed into first-strand cDNA using Reverse Transcription system (Promega, Madison, WI). Quantitative real-time PCR was then performed in duplicate using SYBR premix Ex Taq kit (TaKaRa, Dalian, China) on a DNA Engine Opticon 2 Real-Time PCR Detection System (Bio-Rad, Hercules, CA). Reaction conditions were 95°C for 2 min and then 40 cycles of 95°C for 15 s/60°C for 30 s. The primer sequences are listed in Table 1. Gene expression was normalized to *β*-actin using the ΔΔct method.

### 2.7. Western Blot Analysis

Adipose tissue or cells were lysed in RIPA buffer (50 mM Tris-HCl, pH 7.4, 150 mM NaCl, 1% NP-40, 0.5% sodium deoxycholate, and 0.1% SDS) containing protease and phosphatase inhibitors (5 mM EDTA, 1 mM PMSF, and 1 mM sodium orthovanadate) for 30 min on ice. After centrifugation at 12,000 ×g for 30 min at 4°C, the supernatants were collected and protein concentrations were determined by BCA protein assay (Pierce, Rockford, IL). Equal amounts of protein from each sample were electrophoresed on 10% SDS-PAGE gels and transferred to polyvinylidene difluoride (PVDF) membranes (Millipore, Bedford, MA). The membranes were blocked in 5% skim milk in TBS containing 0.1% Tween-20 (TBST) for 1 h at room temperature and then incubated with different primary antibodies overnight at 4°C. After washing and incubating with HRP-conjugated secondary antibodies for 1 h at room temperature, immunoreactive proteins were visualized using Super Signal Pico ECL reagent (Pierce, Rockford, IL) and exposed to film. To reprobe with different antibodies, the membranes were stripped in stripping buffer containing 62.5 mM Tris-HCl, pH 6.8, 2% SDS, and 100 mM *β*-mercaptoethanol at 50°C for 20–30 min with shaking.

### 2.8. Statistical Analysis

Data are presented as means ± SE unless otherwise stated. Nonnormally distributed data were logarithmically transformed before analysis. Comparisons between groups were carried out using unpaired Student's *t*-test or one-way ANOVA with Bonferroni post hoc test. Correlation analyses were performed using Pearson's test. The cellular experiments were duplicated at least 3 times. All statistical analyses were performed with SPSS 13.0 (Chicago, IL). *P* < 0.05 was considered statistically significant.

## 3. Result

### 3.1. FSTL1 Expression Is Decreased during Differentiation of Preadipocytes

**Figure 1:**
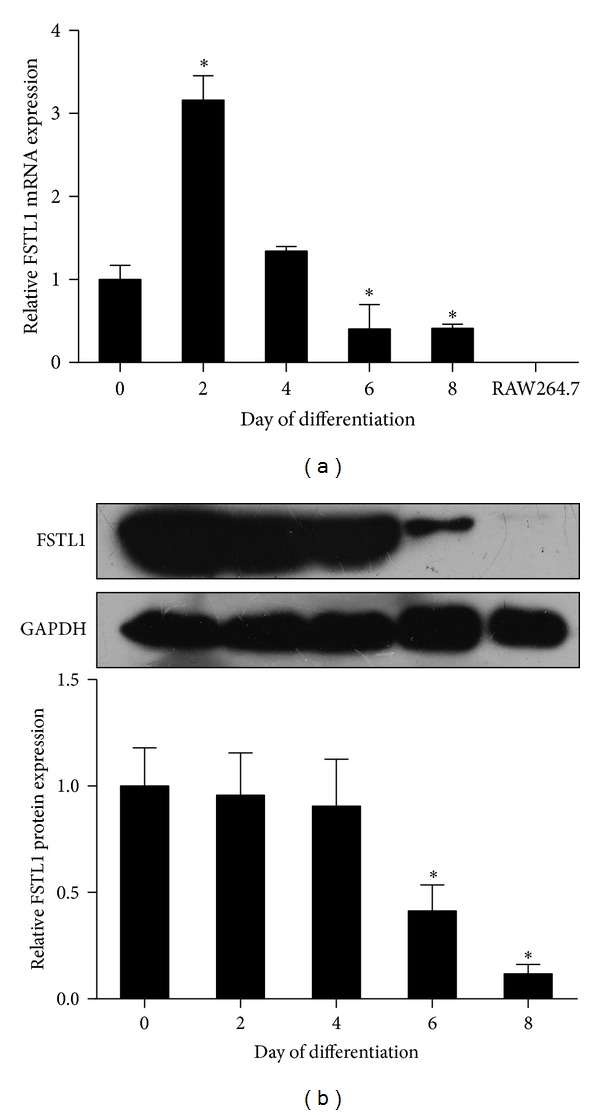
FSTL1 expression is downregulated during differentiation of 3T3-L1 preadipocytes. (a) FSTL1 mRNA expression was evaluated by quantitative RT-PCR in the course of 3T3-L1 preadipocyte differentiation and in RAW264.7 cells. Relative mRNA expression was normalized to *β*-actin and expressed as fold of D0 value. (b) FSTL1 protein expression in differentiating preadipocytes was assessed by Western blot analysis (top) and quantified by densitometry (bottom). Relative protein expression was normalized to GAPDH and expressed as fold of D0 value. Data are mean ± SE; *n* = 3. *P < 0.05 versus undifferentiated preadipocytes (D0).

To explore the role of FSTL1 in adipocytes, we first assessed the expression pattern of FSTL1 upon 3T3-L1 preadipocyte differentiation. Similar to previous study [19], Fstl1 mRNA expression was significantly increased after initiation of preadipocyte differentiation but was markedly decreased afterwards (Figure 1(a)). Consistent with the mRNA results, a striking reduction in FSTL1 protein expression was observed during 3T3-L1 preadipocyte differentiation, though a rise on D2 was not detected (Figure 1(b)). In addition, we also examined the expression of Fstl1 in RAW264.7 macrophages. As shown in Figure 1(a), Fstl1 was rarely detectable in RAW264.7 macrophages.

### 3.2. FSTL1 Expression Is Induced by TNF-*α* in Adipocytes

**Figure 2:**
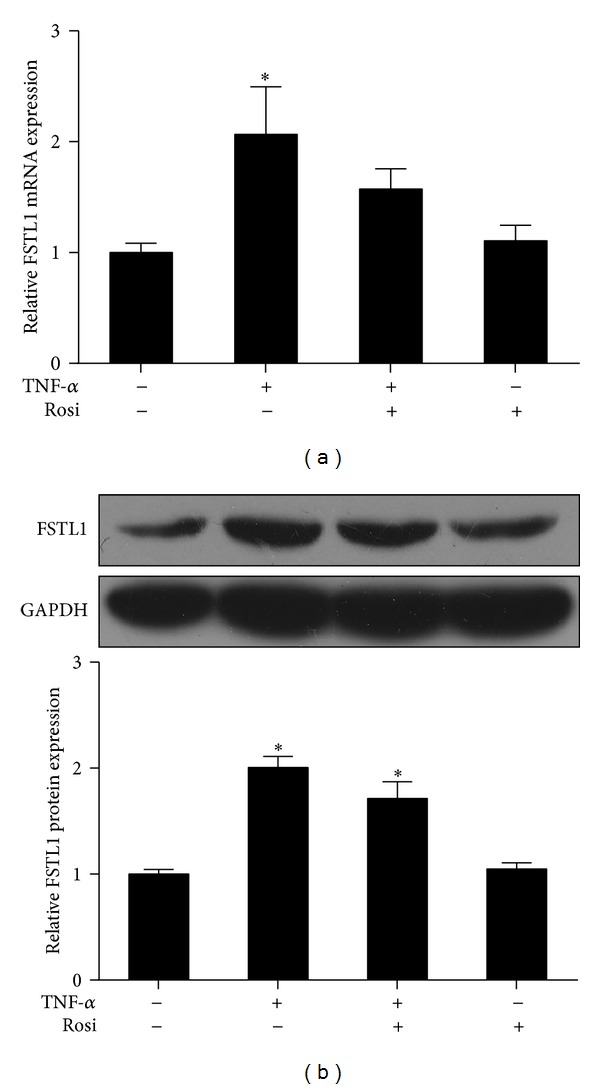
FSTL1 expression is regulated by TNF-*α* in 3T3-L1 adipocytes. Differentiated 3T3-L1 adipocytes were serum-starved for 16 h and then incubated with TNF-*α* (20 ng/mL) for 24 h in the presence or absence of rosiglitazone (Rosi; 1 *μ*M). (a) Fstl1 mRNA expression was evaluated by quantitative RT-PCR and normalized to *β*-actin. Data are expressed as fold of control. (b) FSTL1 protein expression was assessed by Western blot analysis (top) and quantified by densitometry (bottom). Data were normalized to GAPDH and expressed as fold of control. Data are mean ± SE; *n* = 3. *P < 0.05 versus control.

It has been reported that FSTL1 is upregulated by inflammatory factors including TNF-*α* and IL-1*β* in several cell types [24]. To determine whether FSTL1 expression in adipocytes is also modulated by inflammatory stimuli, we treated 3T3-L1 adipocytes with TNF-*α* and/or rosiglitazone, which are well known to promote and attenuate inflammation in adipocytes, respectively [25, 26]. We observed that TNF-*α* led to a significant increase in FSTL1 mRNA and protein expression in adipocytes (Figure 2). Notably, this increase was partially attenuated by coincubation with rosiglitazone (Figure 2). The aforementioned results suggest a potential link between FSTL1 and inflammation in adipocytes.

### 3.3. FSTL1 Expression Is Increased in Adipose Tissues of Obese Mice

**Figure 3:**
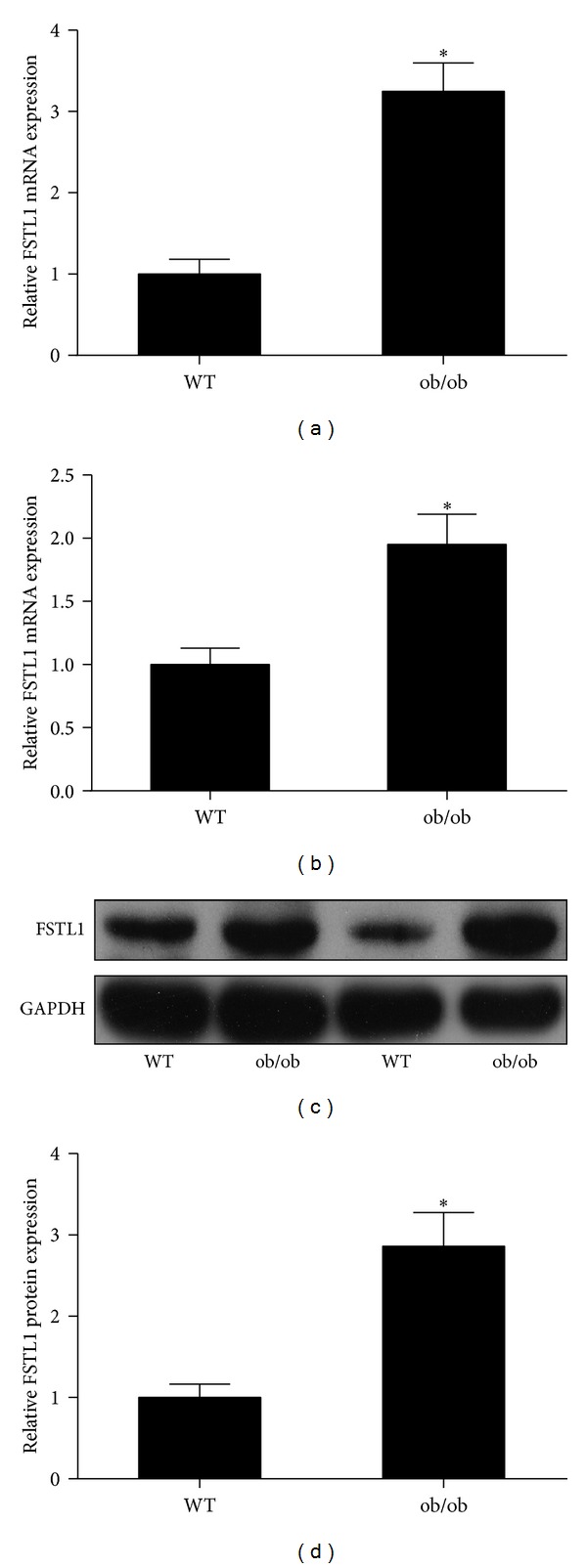
FSTL1 expression is increased in adipose tissue of obese mice. (a) and (b) Fstl1 mRNA expression was evaluated by quantitative RT-PCR in subcutaneous (a) and epididymal (b) adipose tissue of ob/ob mice and littermate controls (*n* = 10 per group). Relative mRNA expression was normalized to *β*-actin and expressed as fold of controls. (c) Western blot analysis of FSTL1 protein expression in epididymal adipose tissue of ob/ob mice and littermate controls (*n* = 4 per group). (d) Intensity of bands was quantified by densitometry. Relative expression was normalized to GAPDH and expressed as fold of controls. *P < 0.05 versus control group.

To probe the role of FSTL1 in vivo, we then examined its expression levels in subcutaneous and epididymal adipose tissue of ob/ob mice, a well-characterized model of severe genetic obesity and insulin resistance resulting from leptin deficiency [27]. As shown in Figures 3(a) and 3(b), Fstl1 mRNA expression levels were markedly increased in both subcutaneous and epididymal adipose tissue of ob/ob mice, as compared with their lean littermate controls. Consistently, epididymal adipose tissue from *ob/ob *mice exhibited a significant upregulation of FSTL1 protein expression when compared with control mice (Figures 3(c) and 3(d)).

### 3.4. Serum FSTL1 Levels Are Associated with Obesity in Humans

In light of our animal results and the evidence that circulating FSTL1 is elevated in patients with systemic inflammatory diseases [18], we further assessed its serum levels in overweight/obese subjects, in whom a state of chronic inflammation is always present. Clinical and biochemical characteristics of the study subjects are shown in Table 2. Serum levels of FSTL1 were significantly higher in overweight/obese subjects than in control subjects after adjusting for age and sex (5.26 ± 1.40 versus 4.14 ± 0.91 ng/mL, *P* = 0.016). Furthermore, a positive correlation between serum FSTL1 levels and BMI was observed and remained significant after adjustment for age and sex (*r* = 0.251, *P* = 0.003). Together, FSTL1 is shown to be associated with obesity in humans.

### 3.5. FSTL1 Induces Inflammatory Mediator Expression in Adipocytes and Macrophages

**Figure 4:**
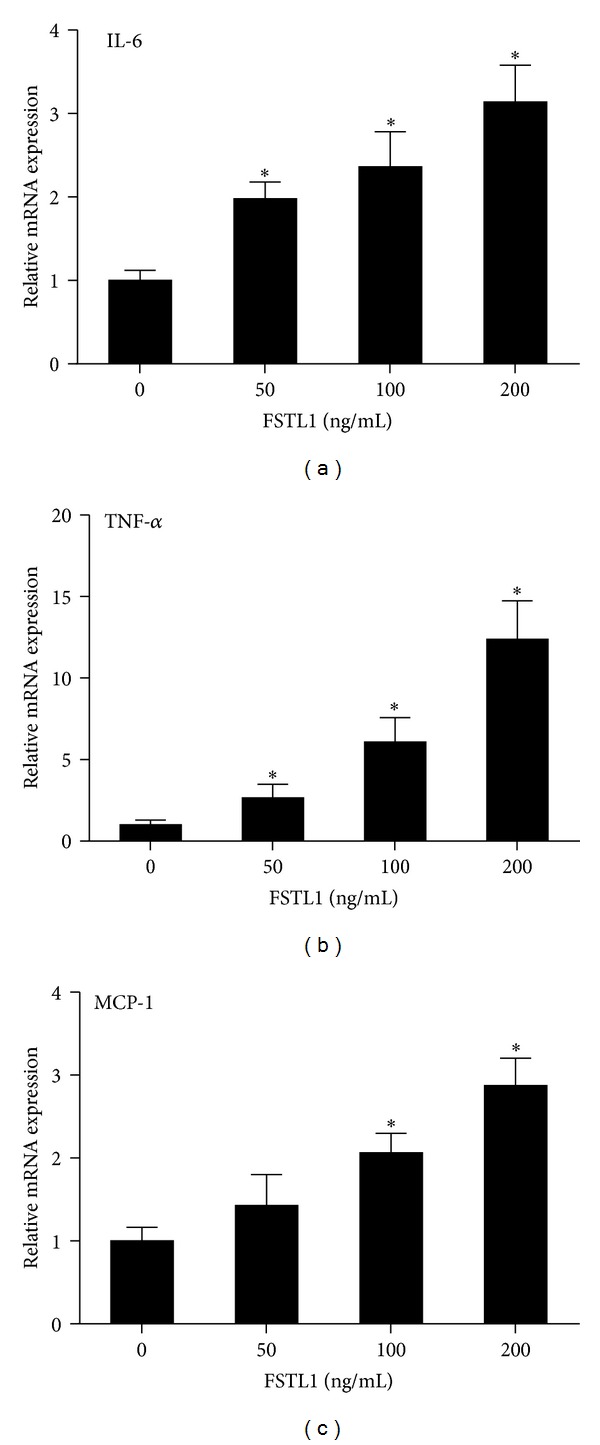
FSTL1 induces the expression of proinflammatory mediators in 3T3-L1 adipocytes. Differentiated 3T3-L1 adipocytes were treated with increasing dose of recombinant mouse FSTL1 (0, 50, 100, and 200 ng/mL) for 24 h. mRNA expressions of IL-6 (a), TNF-*α* (b), and MCP-1 (c) were evaluated by quantitative RT-PCR. Relative gene expression levels were normalized to *β*-actin and expressed as fold of control. Data are mean ± SE; *n* = 3. *P < 0.05 versus control.

We next sought to explore the functional significance of increased FSTL1 expression in obesity. Since FSTL1 has been shown to induce inflammatory mediator expression in several cell types of the mesenchymal and hematopoietic lineage [9, 24], we tested whether FSTL1 exerts a similar effect on adipocytes. 3T3-L1 adipocytes were treated with increasing dose of recombinant mouse FSTL1 and inflammatory mediator expression was assessed by quantitative real-time PCR. We observed that FSTL1 treatment induced IL-6, TNF-*α*, and MCP-1 mRNA expression in 3T3-L1 adipocytes in a dose-dependent manner (Figure 4).

**Figure 5:**
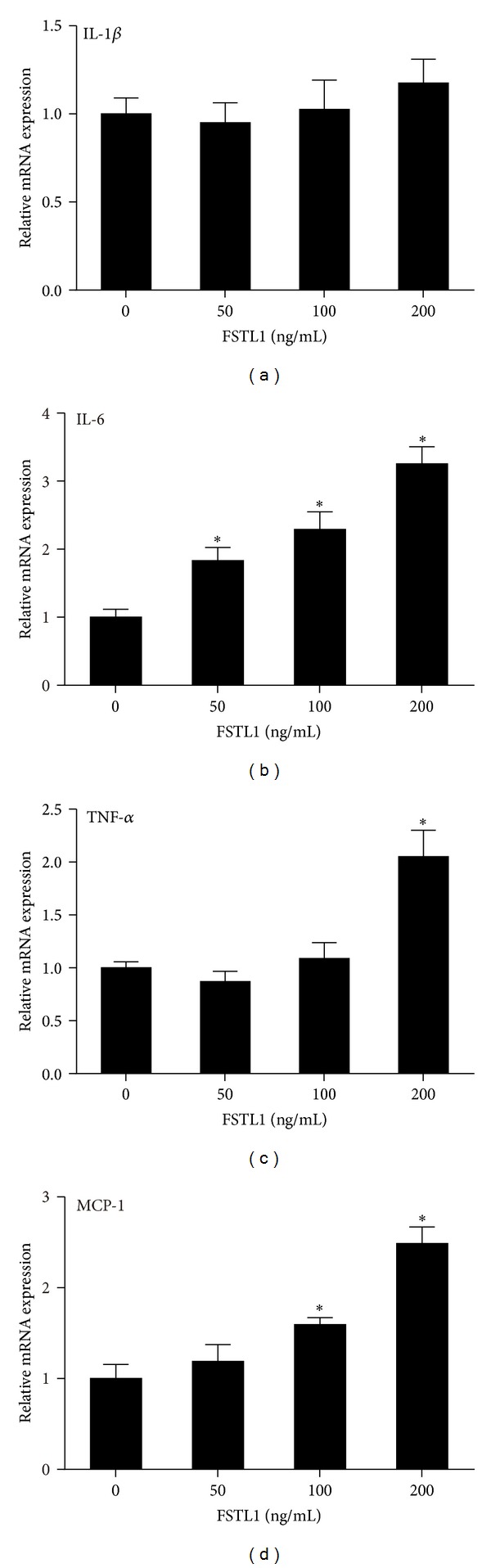
FSTL1 induces the expression of proinflammatory mediators in RAW264.7 macrophages. RAW264.7 macrophages were treated with increasing dose of recombinant mouse FSTL1 (0, 50, 100, and 200 ng/mL) for 24 h. mRNA expressions of IL1-*β* (a), IL-6 (b), TNF-*α* (c), and MCP-1 (d) were evaluated by quantitative RT-PCR. Relative gene expression levels were normalized to *β*-actin and expressed as fold of control. Data are mean ± SE; *n* = 3. *P < 0.05 versus control.

In addition to adipocytes, macrophages are critical in adipose inflammation, and their functions are regulated by signals from preadipocytes/adipocytes [28, 29]. Thus, we further examined the effects of FSTL1 on macrophages. As shown in Figures 5(b) and 5(d), FSTL1 dose-dependently increased IL-6 and MCP-1 mRNA expression in RAW264.7 macrophages. In contrast, FSTL1 stimulated TNF-*α* expression merely at high concentrations (Figure 5(c)) and did not affect IL-1*β* expression (Figure 5(a)). Collectively, our results indicate that FSTL1 has proinflammatory effects on both adipocytes and macrophages.

### 3.6. FSTL1 Activates Inflammatory Signaling Pathways in Adipocytes and Macrophages

**Figure 6:**
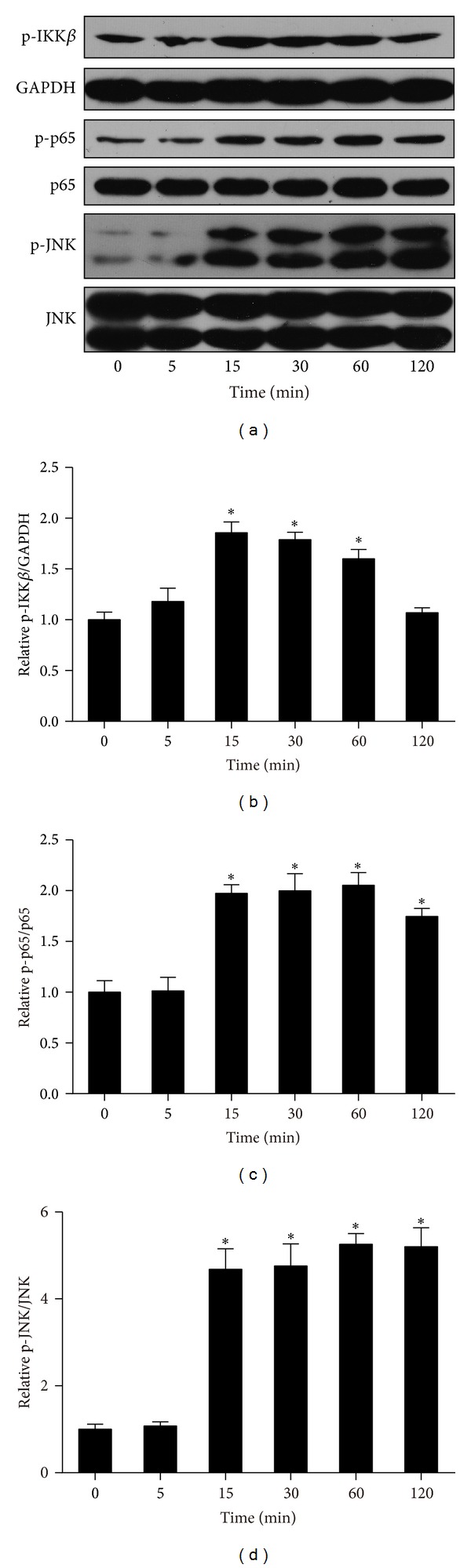
FSTL1 activates proinflammatory signaling pathways in 3T3-L1 adipocytes. Differentiated 3T3-L1 adipocytes were treated with recombinant mouse FSTL1 (100 ng/mL) for indicated time periods (0–2 h). (a) Expression and phosphorylation of IKK*β*, NF*κ*B, and JNK were assessed by Western blot analysis. (b) and (c) Intensity of bands was quantified by densitometry and expressed as ratio of phosphorylated to total protein. The ratio was normalized to control. Data are mean ± SE; *n* = 3. *P < 0.05 versus control (0 min).

**Figure 7:**
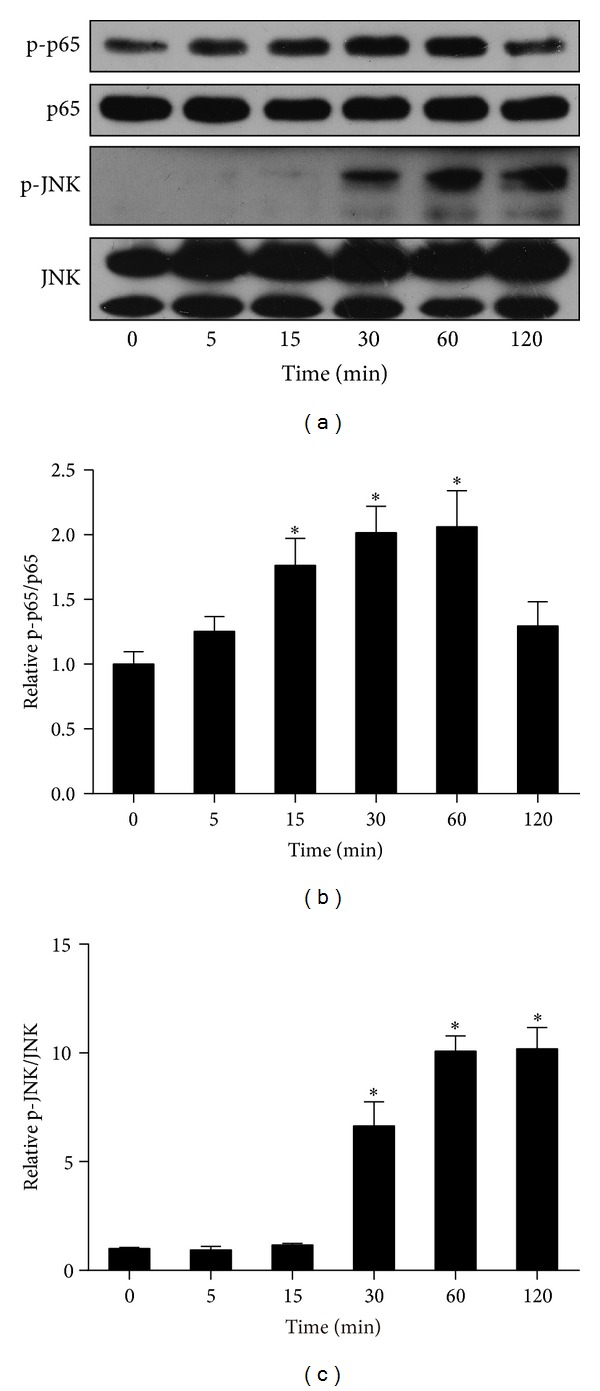
FSTL1 activates proinflammatory signaling pathways in RAW264.7 macrophages. RAW264.7 macrophages were treated with recombinant mouse FSTL1 (100 ng/mL) for indicated time periods (0–2 h). (a) Expression and phosphorylation of NF*κ*B and JNK were assessed by Western blot analysis. (b) and (c) Intensity of bands was quantified by densitometry and expressed as ratio of phosphorylated to total protein. The ratio was normalized to control. Data are mean ± SE; *n* = 3. *P < 0.05 versus control (0 min).

Both NF*κ*B and JNK signaling are critical in obesity-induced inflammation and insulin resistance [30, 31]. Recently, it was reported that FSTL1 activated NF*κ*B signaling in HEK293 cells [21]. To determine whether FSTL1 could stimulate these signaling events in adipocytes, we treated 3T3-L1 adipocytes with recombinant FSTL1 for indicated periods of time. As shown in Figure 6, FSTL1 induced phosphorylation of IKK*β*, NF*κ*B/p65, and JNK as early as 15 min after treatment and up to 60 or 120 min, suggesting that FSTL1 is a direct activator of IKK*β*-NF*κ*B and JNK signaling in adipocytes. We also examined the action of FSTL1 on macrophages. Similarly, FSTL1 activated NF*κ*B and JNK cascades in RAW264.7 macrophages (Figure 7).

### 3.7. FSTL1 Impairs Insulin Signaling in 3T3-L1 Adipocytes

**Figure 8:**
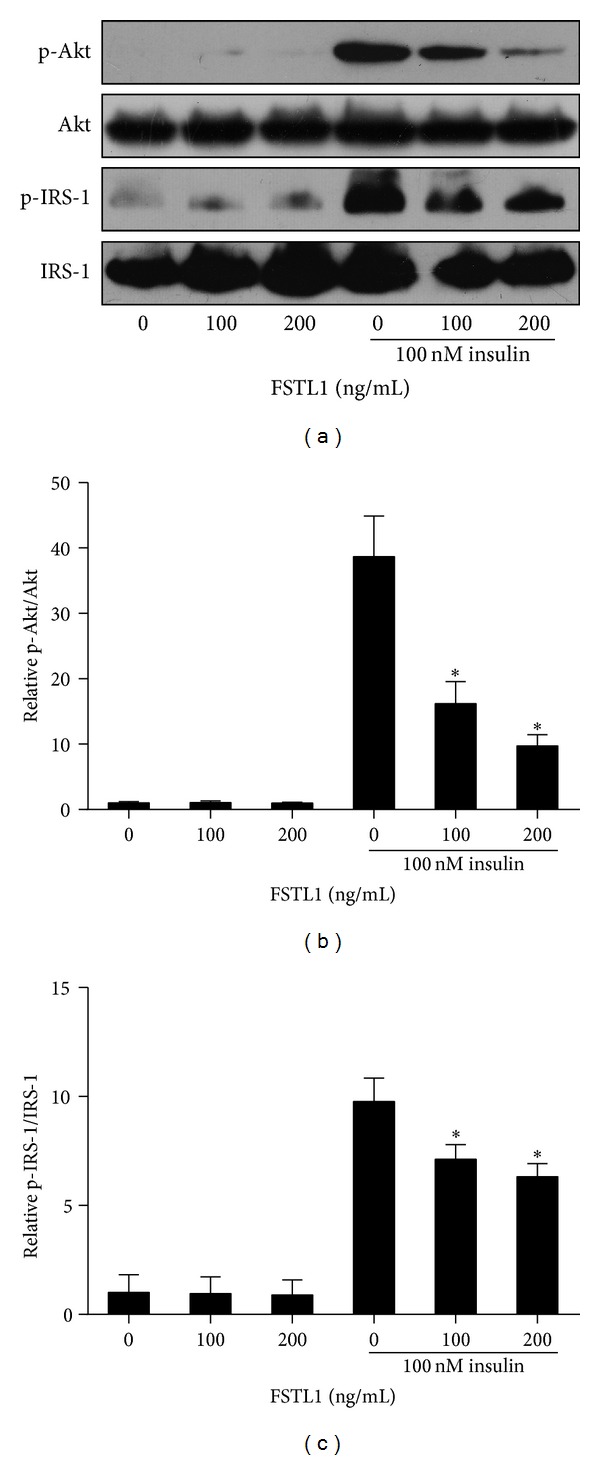
Recombinant FSTL1 inhibits insulin signaling in 3T3-L1 adipocytes. Differentiated 3T3-L1 adipocytes were treated with recombinant mouse FSTL1 (100 or 200 ng/mL) or vehicle for 24 h and subsequently stimulated with 100 nM insulin for 10 min. (a) Phosphorylation of Akt (Ser473) and IRS-1 (Tyr612) was assessed by Western blot analysis. (b) and (c) Intensity of bands was quantified by densitometry and expressed as ratio of phosphorylated to total protein. The ratio was normalized to control. Data are mean ± SE; *n* = 3. *P < 0.05 versus cells only stimulated with insulin.

Given the causative role of inflammation in insulin resistance, we further examined the impact of FSTL1 on insulin signaling in adipocytes. Fully differentiated 3T3-L1 adipocytes were incubated with recombinant FSTL1 for 24 h and then stimulated with insulin (100 nM) for 10 min. As shown in Figures 8(a) and 8(b), insulin-stimulated phosphorylation of Akt on Ser473, a commonly used marker of insulin signaling [32], was markedly reduced by FSTL1 treatment. We then assessed insulin signaling events upstream of Akt. Consistently, FSTL1 decreased specific tyrosine phosphorylation of IRS-1 on Tyr612 in response to insulin stimulation (Figures 8(a) and 8(c)). Taken together, these results indicate that FSTL1 impairs insulin signal transduction in 3T3-L1 adipocytes.

## 4. Discussion

Obesity is associated with chronic inflammation, especially in adipose tissue. However, the underlying mechanisms are not fully understood. In this study, we investigated the association of FSTL1 with obesity and its actions on adipocytes and macrophages. Serum FSTL1 levels were significantly elevated in overweight/obese subjects compared with lean controls. Furthermore, FSTL1 expression was markedly increased in adipose tissues of ob/ob mice, though this genetically obese model may not sufficiently recapitulate the features of obesity in humans. In vitro, recombinant FSTL1 induced proinflammatory mediator expression in both adipocytes and macrophages with activation of NF*κ*B and JNK signaling. Moreover, FSTL1 impaired insulin signaling in adipocytes. Together, our results suggest a potential role of FSTL1 in adipose tissue inflammation and insulin resistance in obesity.

Similar to many proinflammatory mediators, such as IL-6, IL-8, and MCP-1 [33–35], FSTL1 expression was markedly decreased during differentiation of preadipocytes, as seen in our studies and other studies [19]. In contrast, in vivo expression of FSTL1 was significantly upregulated in both subcutaneous and epididymal adipose tissue of obese mice, as well as in serum of overweight/obese subjects. The mechanisms for upregulation of FSTL1 in obese adipose tissue may be multiple and remain to be elucidated. TNF-*α*, which is increased in adipose tissue of obese mice [36], induces FSTL1 expression in adipocytes, as seen in our study, and therefore is a potential candidate for the increase of FSTL1. In addition, TGF-*β*, the first identified factor inducing FSTL1 expression, is also elevated in obese adipose tissue [10, 37] and thus may contribute to the increase of FSTL1 as well. Since FSTL1 is not expressed by cells of the hematopoietic lineage such as macrophages and lymphocytes, as shown in our studies and other studies [24], preadipocytes/adipocytes may be the major source of FSTL1 production in obese adipose tissue. Fractionating the adipose tissue into adipocytes and stromal vascular factions may help determine which faction contributes to the increase of FSTL1 expression in adipose tissue during obesity.

FSTL1 was originally cloned from synovial tissue of rheumatoid arthritis and has been identified as a novel proinflammatory cytokine [9, 38]. It induced the secretion of inflammatory mediators by cells of the mesenchymal lineage such as NIH-3T3 and COS-7 fibroblast, as well as immune cells including U937 monocytes and splenocytes [9, 17, 21]. In vivo, FSTL1 was reported to exacerbate collagen-induced arthritis, associated with enhanced expression of inflammatory cytokines [9]. In contrast, it has also been shown that FSTL1 improved joint inflammation in a model of antibody-induced arthritis [39]. Moreover, Fstl1 overexpression promoted heart allograft survival, associated with reduced expression of inflammatory cytokines, including IL-6 [40]. These discrepancies may result from differences in the experimental models or context-dependent roles of FSTL1 in the regulation of inflammatory responses under various pathological conditions. In our study, we observed proinflammatory activities of FSTL1. Expression of inflammatory mediators, including TNF-*α*, IL-6, and MCP-1, was significantly increased in 3T3-L1 adipocytes and RAW264.7 macrophages stimulated with recombinant FSTL1. Macrophages, a major player in obesity-induced inflammation, are recruited into adipose tissue during obesity and activated by multiple factors, including those from adipocytes [28]. FSTL1 is secreted by adipocytes and can be further enhanced by inflammatory stimuli such as TNF-*α*. In turn, FSTL1 is able to induce inflammatory responses in macrophages. Thus, FSTL1 may provide a link between adipocytes and macrophages in adipose tissue and mediates chronic inflammation in obesity. In addition, FSTL1 could induce inflammation in adipocytes in an autocrine manner, as suggested by our in vitro results. Since FSTL1 levels were increased in adipose tissue of obese mice and in serum of obese subjects, further investigation is warranted to determine whether FSTL1 induces adipose tissue inflammation in vivo.

Both IKK*β*-NF*κ*B and JNK signaling play a critical role in obesity-induced inflammation [41]. Targeted deletion of IKK*β* or JNK protects against inflammation in obese mice [30, 31]. In this study, we observed stimulative effect of FSTL1 on these pathways in both adipocytes and macrophages. FSTL1 potently activated IKK*β*-NF*κ*B and JNK as early as 15 min after treatment, suggesting a direct action on them. Indeed, FSTL1 has been shown to activate NF*κ*B using its reporter system in HEK293 cells [21]. Thus, it may be through IKK*β*-NF*κ*B and JNK signaling that FSTL1 induces inflammatory response in adipocytes and macrophages.

In contrast to the studies focusing on the functions of FSTL1, the underlying mechanisms are poorly understood. In the present study, FSTL1 activated inflammatory pathways in adipocytes and macrophages. However, the mechanisms by which FSTL1 stimulates these pathways remain unresolved. Recently, FSTL1 was shown to activate TLR4 signaling [21]. TLR4 is a pattern recognition receptor that recognizes invading pathogens and triggers inflammatory responses [42]. Besides, TLR4 also detects many endogenous molecules such as saturated fatty acid and induces sterile inflammation in a range of chronic diseases, including obesity and type 2 diabetes [43]. Given the abundant expression of TLR4 in adipocytes and macrophages [44, 45], further studies are warranted to determine whether FSTL1 induces inflammatory responses in these cells via activating TLR4 signaling.

Chronic inflammation plays a central role in the development of insulin resistance [2]. In light of the proinflammatory property of FSTL1, we examined its impact on insulin sensitivity. As revealed by reduced phosphorylation of Akt and IRS-1 in response to insulin, FSTL1 impaired insulin signaling in adipocytes. Nevertheless, the mechanisms whereby FSTL1 interacts with insulin signaling remain undetermined. We detected immediate activation of IKK*β*-NF*κ*B and JNK pathways in 3T3-L1 adipocytes after FSTL1 treatment. It has been demonstrated that IKK*β* and JNK can phosphorylate IRS-1 on serine residues, which in turn ablates tyrosine phosphorylation of IRS-1 and thereby reduces insulin signaling [46–48]. Thus, there is good reason to assume that FSTL1 could attenuate insulin signaling via direct activation of IKK*β* and JNK. Alternatively, other cytokines, such as TNF-*α* and IL-6, were induced by FSTL1 and therefore may also mediate the effect of FSTL1 on insulin sensitivity. The relative contribution of direct and indirect actions of FSTL1 to impaired insulin signaling in adipocytes remains to be determined. On the other hand, to further confirm the suppressive effect of FSTL1 on insulin signaling, in vivo studies are needed. For instance, correlation analysis between FSTL1 expression in adipose tissue and the development of insulin resistance in ob/ob mice may provide evidence for its implication in insulin resistance. In addition, adenovirus-mediated overexpression of FSTL1 or blocking its actions through neutralizing antibodies in mice, as used in previous studies [9, 17], will more directly elucidate the role of FSTL1 in the pathogenesis of insulin resistance.

## 5. Conclusion

In the present study, we show that FSTL1 is associated with obesity in both mice and humans. It induces inflammatory responses in adipocytes and macrophages and suppresses insulin signaling in adipocytes. However, further research is required to elucidate the role of FSTL1 in the development of inflammation and insulin resistance in vivo.

## Figures and Tables

**Table 1:** Primer sequences for real-time PCR.

Genes	Sense	Antisense
Fstl1	CACGGCGAGGAGGAACCTA	TCTTGCCATTACTGCCACACA
IL-1*β*	CAACCAACAAGTGATATTCTCCATG	GATCCACACTCTCCAGCTGCA
IL-6	GAGGATACCACTCCCAACAGACC	AAGTGCATCATCGTTGTTCATACA
TNF-*α*	CATCTTCTCAAAATTCGAGTGACAA	TGGGAGTAGACAAGGTACAACCC
MCP-1	CTTCTGGGCCTGCTGTTCA	CCAGCCTACTCATTGGGATCA
*β*-Actin	GGCTGTATTCCCCTCCATCG	CCAGTTGGTAACAATGCCATGT

**Table 2:** Clinical and biochemical characteristics of the study subjects.

Characteristics	Normal weight	Overweight/obese	*P* value
Number of subjects	93	51	
Age (years)	45.5 ± 1.6	48.5 ± 1.8	0.244
Male, *n* (%)	25 (27)	31 (60)	<0.001
BMI (kg/m^2^)	22.07 ± 0.18	26.45 ± 0.18	<0.001
SBP (mmHg)	117.0 ± 1.6	126.3 ± 2.7	0.002
DBP (mmHg)	73.7 ± 1.0	78.7 ± 1.4	0.003
FBG (mmol/L)	4.76 ± 0.04	4.77 ± 0.05	0.888
TG (mmol/L)	1.18 ± 0.08	1.90 ± 0.18	<0.001
TC (mmol/L)	4.79 ± 0.10	4.90 ± 0.12	0.501
LDL-C (mmol/L)	3.01 ± 0.10	3.12 ± 0.11	0.490
HDL-C (mmol/L)	1.48 ± 0.03	1.29 ± 0.05	0.002

Data are presented as number (percentage) for categorical data and mean ± SE for continuous data.
